# Comparison of the ligand‐binding properties of fluorescent VEGF‐A isoforms to VEGF receptor 2 in living cells and membrane preparations using NanoBRET

**DOI:** 10.1111/bph.14755

**Published:** 2019-07-15

**Authors:** Chloe J. Peach, Laura E. Kilpatrick, Jeanette Woolard, Stephen J. Hill

**Affiliations:** ^1^ Division of Physiology, Pharmacology and Neuroscience, School of Life Sciences University of Nottingham Nottingham UK; ^2^ Centre of Membrane Proteins and Receptors University of Birmingham and University of Nottingham The Midlands UK

## Abstract

**Background and Purpose:**

Vascular endothelial growth factor A (VEGF‐A) is a key mediator of angiogenesis. A striking feature of the binding of a fluorescent analogue of VEGF_165_a to nanoluciferase‐tagged VEGF receptor 2 (VEGFR2) in living cells is that the BRET signal is not sustained and declines over time. This may be secondary to receptor internalisation. Here, we have compared the binding of three fluorescent VEGF‐A isoforms to VEGFR2 in cells and isolated membrane preparations.

**Experimental Approach:**

Ligand‐binding kinetics were monitored in both intact HEK293T cells and membranes (expressing nanoluciferase‐tagged VEGFR2) using BRET between tagged receptor and fluorescent analogues of VEGF_165_a, VEGF_165_b, and VEGF_121_a. VEGFR2 endocytosis in intact cells expressing VEGFR2 was monitored by following the appearance of fluorescent ligand‐associated receptors in intracellular endosomes using automated quantitative imaging.

**Key Results:**

Quantitative analysis of the effect of fluorescent VEGF‐A isoforms on VEGFR2 endocytosis in cells demonstrated that they produce a rapid and potent translocation of ligand‐bound VEGFR2 into intracellular endosomes. NanoBRET can be used to monitor the kinetics of the binding of fluorescent VEGF‐A isoforms to VEGFR2. In isolated membrane preparations, ligand‐binding association curves were maintained for the duration of the 90‐min experiment. Measurement of the *k*_off_ at pH 6.0 in membrane preparations indicated shorter ligand residence times than those obtained at pH 7.4.

**Conclusions and Implications:**

These studies suggest that rapid VEGF‐A isoform‐induced receptor endocytosis shortens agonist residence times on the receptor (1/*k*_off_) as VEGFR2 moves from the plasma membrane to the intracellular endosomes.

**Abbreviations**
RTK: receptor TK
RTKI: receptor TK inhibitor
TMR: tetramethylrhodamine

**What is already known**

- VEGF‐A is a key mediator of angiogenesis.
- The binding of VEGF‐A to VEGFR2 is not sustained in living cells.

**What this study adds**

- The power of NanoBRET approaches to study real‐time ligand‐binding kinetics in membranes and cells.
- Fluorescent VEGF‐A isoforms produced a rapid and potent translocation of ligand‐bound VEGFR2 into intracellular endosomes.
- Endocytosis shortens agonist residence times as VEGFR2 moves from the plasma membrane to intracellular endosomes.

**What is the clinical significance**

- New insights into the impact of cellular location on the kinetics of VEGFR2 ligand–receptor interactions.

## 1 INTRODUCTION

Angiogenesis, the growth of new blood vessels from existing vascular networks, is an important physiological process that can be dysregulated in numerous pathologies including cancer and age‐related macular degeneration (Chung & Ferrara, 2011; Peach, Mignone, et al., 2018). VEGF‐A is a key mediator of both angiogenesis and vascular permeability, primarily signalling via its cognate receptor VEGF receptor 2 (VEGFR2; Alexander et al., 2017; Simons, Gordon, & Claesson‐Welsh, 2016). VEGF‐A is an anti‐parallel, disulphide‐linked homodimer that binds across immunoglobulin‐like domains 2 and 3 of VEGFR2 (Ruch, Skiniotis, Steinmetz, Walz, & Ballmer‐Hofer, 2007). Ligand binding leads to VEGFR2 dimerisation and conformational changes that enable the auto‐ and trans‐phosphorylation of intracellular tyrosine residues. The recruitment of adaptor proteins to VEGFR2 and subsequent initiation of signalling cascades then lead to cell proliferation, migration, and vascular permeability (Koch, Tugues, Li, Gualandi, & Claesson‐Welsh, 2011; Simons et al., 2016). VEGFR2 can internalise in the presence or absence of ligand stimulation (Ewan et al., 2006; Jopling et al., 2009; Jopling, Howell, Gamper, & Ponnambalam, 2011). Through both clathrin‐dependent and clathrin‐independent mechanisms (Basagiannis et al., 2016), VEGFR2 first accumulates in Rab5+/EEA1+ endosomes (Gampel et al., 2006). VEGFR2 can then recycle back to the plasma membrane via short loop Rab4+ endosomes or long loop Rab11+ endosomes (Jopling et al., 2014), or receptor ubiquitination can initiate lysosomal degradation (Smith et al., 2016).

Alternative mRNA splicing of the *Vegfa* gene leads to a number of endogenous VEGF‐A isoforms of varying length, such as “prototypical” VEGF_165_a or VEGF_121_a, as well as variants (e.g., VEGF_165_b) that have distinct carboxy‐terminus substitutions of exon 8a for exon 8b (Peach, Mignone, et al., 2018; Woolard et al., 2004). Additionally, programmed translational readthrough can lead to VEGF‐Ax, an isoform containing both exon 8a‐ and 8b‐encoded residues (Eswarappa et al., 2014). The residues present within each VEGF‐A isoform determine whether they can interact with other membrane proteins (e.g., neuropilin 1; Cébe Suarez et al., 2006; Parker, Xu, Li, & Vander Kooi, 2012; Guo & Vander Kooi, 2015; Peach, Kilpatrick, et al., 2018) and extracellular matrix components (Krilleke et al., 2007; Vempati, Popel, & Mac Gabhann, 2014). This causes isoforms to vary in their bioavailability and signalling outcomes with many isoforms acting as partial agonists relative to VEGF_165_a (Peach, Mignone, et al., 2018). VEGF‐A isoforms also have distinct expression profiles in health and disease, such as down‐regulation of VEGF_165_b in numerous cancer types (Bates et al., 2002; Pritchard‐Jones et al., 2007). VEGF‐A/VEGFR2 signalling has been targeted by a number of clinically approved inhibitors used to treat cancer, such as receptor TK inhibitors (RTKIs) that target the intracellular ATP‐binding domain (Ferrara & Adamis, 2016).

The development of fluorescence‐based technologies has advanced our pharmacological understanding of GPCRs, RTKs, and other classes of membrane proteins (Stoddart, Kilpatrick, & Hill, 2018; Stoddart, White, Nguyen, Hill, & Pfleger, 2016). For example, real‐time ligand binding can be quantified in living cells using BRET (Stoddart et al., 2015; Stoddart et al., 2016). This proximity‐based assay monitors energy transfer between a receptor tagged on its N‐terminus with a bright 19‐kDa nanoluciferase (NanoLuc) and a suitable fluorophore acceptor. We previously developed fluorescent variants of VEGF_165_a, VEGF_165_b, and VEGF_121_a labelled at a single site with tetramethylrhodamine (TMR; Kilpatrick et al., 2017; Peach, Kilpatrick, et al., 2018). Despite similar affinities, VEGF_165_a‐TMR had distinct binding kinetics at VEGFR2 and its co‐receptor neuropilin 1 expressed in living HEK293T cells (Peach, Kilpatrick, et al., 2018). VEGF_121_a‐TMR and VEGF_165_b‐TMR were also shown to bind to VEGFR2 but not to neuropilin 1 using both NanoBRET and live‐cell fluorescence imaging techniques (Peach, Kilpatrick, et al., 2018).

A striking feature of the binding of VEGF_165_a‐TMR to VEGFR2 in intact living cells is that the BRET signal obtained with higher concentrations of the fluorescent probe declines over longer incubation times, after reaching a peak between 15 and 20 min (Kilpatrick et al., 2017). One possible explanation is that this is a consequence of receptor internalisation and uncoupling of ligand–receptor complexes within intracellular endosomes (Kilpatrick et al., 2017). Due to the complex spatiotemporal dynamics of VEGFR2, kinetic profiles of ligand binding to VEGFR2 in intact living cells are likely to contain components that represent the initial ligand‐binding interaction and also components that reflect receptor endocytosis, whereby the endosomal environment can impact upon the stability of these ligand–receptor complexes.

In order to isolate the ligand‐binding profiles of fluorescent VEGF‐A isoforms to VEGFR2 from the potential influences of agonist‐induced receptor endocytosis, the present study was undertaken with VEGF_165_a‐TMR, VEGF_165_b‐TMR, and VEGF_121_a‐TMR to: (a) investigate the concentration‐dependence and temporal profile of ligand‐induced VEGFR2 endocytosis, (b) the influence of VEGFR2 phosphorylation on endocytosis and ligand binding, and (c) the kinetics of the ligand–receptor interactions in isolated membrane preparations, where the potential for parallel receptor endocytosis is not present.

## 2 METHODS

### 2.1 Cell culture and materials

HEK293T cells (CCLV Cat# CCLV‐RIE 1018, RRID:CVCL_0063) were maintained at 37°C/5% CO_2_ in DMEM (Sigma‐Aldrich, USA) supplemented with 10% fetal calf serum (FCS; Sigma‐Aldrich, USA). At 70–80% confluency, cells were passaged using PBS (Lonza, Switzerland) and trypsin (0.25% w/v in versene; Lonza). Stable cell lines expressing VEGFR2 were generated using FuGENE HD (Promega Corporation, USA) at a 3:1 ratio of reagent to cDNA. As described previously by Peach, Kilpatrick, et al. (2018), N‐terminal NanoLuc‐tagged VEGFR2 (NM_002253) was cloned into a pFN31K vector encoding the secretory IL‐6 signal peptide fused to the N‐terminus of NanoLuc, followed by a GSSGAIA linker before the receptor. Additionally, VEGFR2 was cloned into a pFN21A vector with the IL‐6 signal peptide followed by a sequence encoding HaloTag and an EPTTEDLYFQSDNAIA linker at the receptor N‐terminus, as described in Peach, Kilpatrick, et al. (2018). Fluorescent VEGF_165_a, VEGF_165_b, and VEGF_121_a were labelled at a single N‐terminal cysteine residue with TMR using the HaloTag mammalian protein detection and purification system (G6795; Promega Corporation, USA) as described previously (Kilpatrick et al., 2017; Peach, Kilpatrick, et al., 2018). Fluorescent ligands were characterised in terms of labelling efficiency, dimerisation, and function as described in Kilpatrick et al. (2017) and Peach, Kilpatrick, et al. (2018). Binding affinities of fluorescent VEGF isoforms are approximately an order of magnitude lower than those of their native counterparts (Peach, Kilpatrick, et al., 2018). Ligands were stored at −20°C in 2.5 mg·ml^−1^ protease‐free BSA (Millipore, USA). Cediranib was purchased from Sequoia Research Products (Pangbourne, UK), and unlabelled recombinant human VEGF isoforms were purchased from R&D Systems (Abingdon, UK). Furimazine was bought from Promega Corporation (Madison, USA), and other tissue culture reagents were purchased from Sigma‐Aldrich (Gillingham, UK).

### 2.2 Generation of a tyrosine phosphorylation‐deficient variant of HaloTag‐VEGFR2 and NanoLuc‐VEGFR2

To generate tyrosine phosphorylation‐deficient (VEGFR2‐TPD) variants, site‐directed mutagenesis was performed for HaloTag‐VEGFR2 and NanoLuc‐VEGFR2 using the following forward and reverse complementary primers for mutations: Y951F (Fwd 5′‐GTCAAGGGAAAGACTtCGTTGGAGCAATCCC‐3′; Rev 5′‐GGGATTGCTCCAACGaAGTCTTTCCCTTGAC‐3′), Y1175F (Fwd 5′‐GCAGGATGGCAAAGACTtCATTGTTCTTCCGATATC‐3′; Rev 5′‐GATATCGGAAGAACAATGaAGTCTTTGCCATCCTGC‐3′), Y1214F (Fwd 5′‐GTGACCCCAAATTCCATTtTGACAACACAGCAGGAATC‐3′; Rev 5′‐GATTCCTGCTGTGTTGTCAaAATGGAATTTGGGGTCAC‐3′), and Y1054F (Fwd 5′‐GCTTGGCCCGGGATATTTtTAAAGATCCAGATTATGTC‐3′; Rev 5′‐GACATAATCTGGATCTTTAaAAATATCCCGGGCCAAGC‐3′), and Y1059F was prepared after the Y1054F mutation had been incorporated (shown in bold below; Fwd 5′‐GATATTT**T**TAAAGATCCAGATTtTGTCAGAAAAGGAGATGCTCGC‐3′; Rev 5′‐GCGAGCATCTCCTTTTCTGACAaAATCTGGATCTTTA**A**AAATATC‐3′).

The altered nucleotides are shown in lower case. Mutagenesis was performed sequentially with the above primers to generate the Y951F, Y1054, Y1059F, Y1175F, and Y1214F tyrosine phosphorylation‐deficient mutant of VEGFR2 (VEGFR2‐TPD; Figure 4d) using Pfu DNA polymerase (Promega Corporation, USA), followed by sequencing of the full plasmid to check for off‐target SNPs. All HEK293T cells also stably expressed a Firefly luciferase reporter downstream of an NFAT response element to monitor NFAT‐induced gene transcription (NFAT‐RE‐luc2P; Promega Corporation, USA), as in Carter, Wheal, Hill, and Woolard (2015).

### 2.3 Membrane preparations

HEK293T cells stably expressing wild‐type or tyrosine phosphorylation‐deficient NanoLuc‐VEGFR2 were grown in DMEM/10% FCS to 80–90% confluency in 148‐mm^2^ culture dishes (Corning, USA). The media was then replaced with PBS, cells were removed from the dish by scraping and then transferred into a 50‐ml tube. Cells were centrifuged at 378 × *g* for 12 min at 4°C, the supernatant was removed, and the remaining pellet was stored at −80°C. Thawed pellets resuspended in PBS were homogenised using an electronic handheld IKA T10 Ultra Turrax homogeniser in 10 × 3 s bursts at 15,000 rpm. Unbroken cells and nuclei were removed by centrifugation at 1,500 × *g* for 20 min (4°C). The supernatant was then centrifuged at 41,415 × *g* for 30 min at 4°C to pellet the remaining membranes. The pellet was resuspended in 1‐ml PBS, transferred to a borosilicate glass homogeniser mortar, and homogenised 15 times using an IKA RW16 overhead stirrer attached to a serrated pestle (Kartell) at 1,000 rpm. Protein concentration was determined using a bicinchoninic acid assay (Pierce™ BCA Protein Assay; Thermo Fisher Scientific), and absorbance was measured using a Dynex Technologies 4.25 platereader. Membrane preparations were stored at −80°C, and retained their luminescence emissions following addition of furimazine and their ability to bind VEGF‐TMR for at least 10 months.

### 2.4 Measuring ligand binding using NanoBRET

For whole‐cell experiments with wild‐type VEGFR2, HEK293T cells stably expressing full‐length NanoLuc‐VEGFR2 were grown to 70–80% confluency. Cells were seeded in DMEM/10% FCS at 35,000 cells per well on white 96‐well clear bottom plates (Greiner Bio‐One, 655089) pre‐coated with poly‐D‐lysine (0.01 mg·ml^−1^ in PBS). Following a 24‐hr incubation at 37°C/5% CO_2_, media were replaced with assay buffer, HEPES buffered saline solution (HeBSS; 10 mM HEPES, 10mM glucose, 146 mM NaCl, 5mM KCl, 1 mM MgSO_4_, 2 mM sodium pyruvate, 1.3 mM CaCl_2_; pH 7.2) containing 0.1% BSA at pH 7.4. In transient transfection experiments, HEK293T cells were plated at 12,500 cells per well in white 96‐well clear bottom plates (37°C, 5% CO_2_). After 24 hr, cells were transiently transfected with wild‐type or tyrosine phosphorylation‐deficient NanoLuc‐VEGFR2 using FuGENE HD (Promega Corporation, USA) at a 3:1 ratio of reagent to cDNA with 100‐ng cDNA per well. For assays using membrane preparations derived from stable cell lines, defrosted membranes were plated in white 96‐well clear bottom plates at concentrations ranging from 2.5 to 20 μg per well diluted in HeBSS/0.1% BSA and then incubated for 30 min at 37°C.

For experiments investigating the influence of membrane concentration on NanoBRET signals, increasing concentrations of membranes prepared from wild‐type VEGFR2 cells were incubated in the presence and absence of 5‐nM VEGF_165_b‐TMR. Following incubation for 60 min in the dark, the NanoLuc substrate furimazine (10 μM) was added to each well and equilibrated for 5 min to enable NanoLuc‐mediated furimazine oxidation and resulting bioluminescence emission. Emissions were recorded using the PHERAstar FS platereader (BMG Labtech), and BRET ratios were calculated as fluorescence over luminescence emissions from the second of three cycles.

For kinetic experiments, wells were pretreated with the NanoLuc substrate, furimazine (10 μM), for 5 min to enable NanoLuc‐mediated furimazine oxidation and resulting bioluminescence emission. BRET ratios were then measured per well using the PHERAstar FS platereader (BMG Labtech) using filters measuring NanoLuc emissions at 450 nm (30‐nm bandpass) and TMR emissions using a longpass filter at 550 nm. Following four initial measurements, intact cells were stimulated with two concentrations of VEGF_165_b‐TMR or VEGF_121_a‐TMR at a concentration around the *K*_*D*_ (3 nM) and at 20 nM as a saturating concentration. BRET ratios were then calculated every 30 s for up to 120 min. In contrast, kinetic experiments in membranes used 10 μg per well of membranes, and five concentrations of each fluorescent VEGF‐A isoform (1–20 nM) were used to calculate kinetic binding parameters at wild‐type VEGFR2. To investigate the influence of VEGFR2 phosphorylation on ligand‐binding kinetics at NanoLuc‐VEGFR2, corresponding cells or membranes were pre‐incubated with 1‐μM cediranib (Sequoia Research Products), an RTKI that targets the intracellular ATP‐binding domain, or 0.01% DMSO in the HeBSS/0.1% BSA assay buffer for 30 min at 37°C. Alternatively, kinetics were quantified at tyrosine phosphorylation‐deficient NanoLuc‐VEGFR2 (VEGFR2‐TPD) membranes prepared from a stable cell line. Kinetic experiments were then performed with four concentrations of VEGF_165_b‐TMR (cediranib experiments) or VEGF_121_a‐TMR (VEGFR2‐TPD experiments) for 90 min (3–20 nM). Emissions were recorded every 30 s for 90 min, using the temperature control function of the PHERAstar FS platereader to maintain conditions at 37°C.

For saturation and displacement experiments, membrane preparations were used at 5 μg per well. Increasing concentrations of fluorescent VEGF‐A isoforms were added in the presence or absence of a high concentration of corresponding unlabelled ligand (100 nM, ~100‐fold greater than the estimated *K*_*D*_ from Peach, Kilpatrick, et al., 2018). In displacement experiments, membranes were co‐stimulated with five fixed concentrations of VEGF_165_a‐TMR (0.25−3 nM), VEGF_165_b‐TMR, or VEGF_121_a‐TMR (0.5–5 nM) in the presence of vehicle or increasing concentrations of VEGF‐Ax (0.03–30 nM; R&D Systems). Following a 60‐min incubation (37°C), 10‐μM furimazine was added to each well and equilibrated for 5 min. Emissions were recorded using the PHERAstar FS platereader (BMG Labtech), and BRET ratios were calculated from the second of three cycles.

Both kinetic and saturation experiments were repeated in wild‐type NanoLuc‐VEGFR2 membranes to investigate the effect of pH on ligand binding, using VEGF_165_b‐TMR as a representative fluorescent VEGF‐A isoform. The pH of the assay buffer, HeBSS/0.1% BSA (pH 7.4), was lowered to 5.8–6.2 with concentrated hydrochloric acid on the day of the experiment and measured using a pH Meter PB‐11 (Sartorius, Germany). Although EPES buffer has a pK_a_ suited to physiological pH (6.8–8.2), control experiments confirmed the assay buffer remained within 0.2 of the initial pH in conditions replicating the experimental set‐up (50 μl at 37°C for 2 hr).

### 2.5 High‐content widefield imaging quantifying endocytosis of fluorescent VEGF‐A isoforms

HEK293T cells stably expressing HaloTag‐VEGFR2, NanoLuc‐VEGFR2 (Kilpatrick et al., 2017), or wild‐type HEK293T cells were seeded at 25,000 cells per well in DMEM/10% FCS on black 96‐well clear bottom plates (Greiner Bio‐One, 655090) pre‐coated with poly‐D‐lysine (0.01 mg·ml^−1^ in PBS). Following an incubation for 24 hr at 37°C/5% CO_2_, media were replaced with assay buffer (serum‐free DMEM/0.1% BSA). To compare untransfected HEK293T cells with cells expressing HaloTag‐VEGFR2, cells were stimulated with 10‐nM VEGF_165_b‐TMR and VEGF_121_a‐TMR for 60 min (37°C). All other widefield imaging assays used cells expressing NanoLuc‐VEGFR2. For concentration–response experiments, increasing concentrations of VEGF_165_a‐TMR, VEGF_165_b‐TMR, or VEGF_121_a‐TMR (0.1–100 nM) were added in duplicate wells (60 min; 37°C/5% CO_2_). Alternatively, cells were stimulated with 10‐nM VEGF_165_a‐TMR, VEGF_165_b‐TMR, or VEGF_121_a‐TMR for between 5 and 120 min as a retrospective timecourse. Cells were washed with PBS (100 μl per well) and fixed with 3% paraformaldehyde in PBS for 15 min at room temperature (RT). Following another PBS wash, nuclei were stained with 2 mg·ml^−1^ H33342 in PBS for 15 min at RT and then washed and stored in PBS at 4°C. The following day, cells were imaged using an ImageXpress Micro widefield high‐content platereader (Molecular Devices, USA). Plates were imaged at four sites per well using a 20× ELWD (extra long working distance) objective, with a TRITC filter to image VEGF_xxx_x‐TMR (560‐nm excitation, 607‐ to 634‐nm emission, and 2,000‐ms exposure time) and a DAPI filter for nuclei (405‐nm excitation, 447‐ to 460‐nm emission, and 25‐ms exposure time). Images were analysed using a granularity algorithm (Molecular Devices), whereby nuclei were identified based upon their size (5‐ to 25‐μm diameter) and pixel depth in grey levels, which was kept consistent between experimental replicates. A nuclear mask defining cell nuclei was then placed over the nuclei within the acquired image forming the basis for automated image segmentation. Fluorescent granules were defined based upon size (2‐ to 15‐μm diameter) and pixel depth in grey levels. Granules were then assigned to specific nuclei based upon proximity using the aforementioned segmented image.

### 2.6 Immunofluorescent labelling of Rab5

HEK293T cells stably expressing wild‐type or tyrosine phosphorylation‐deficient HaloTag‐VEGFR2 were seeded at 300,000 cells per well onto poly‐D‐lysine‐coated coverslips (18 × 18 mm, 1.5H; Zeiss, Germany) in six‐well plates. Following a 24 hr incubation at 37°C/5% CO_2_, coverslips were transferred to humidified wells of a six‐well plate lined with parafilm and maintained in PBS to retain moisture. Receptors were labelled with 0.5‐μM membrane‐impermeant HaloTag‐Alexa Fluor 647 (Promega Corporation, USA) in assay buffer (serum free DMEM containing 0.1% BSA). Following 30 min at 37°C/5% CO_2_, coverslips were washed twice with assay buffer and then incubated with 10‐nM VEGF_121_a‐TMR for 5 or 60 min (37°C/5% CO_2_). Cells were washed with PBS and fixed with 3% paraformaldehyde in PBS for 20 min at RT. Following numerous wash steps in PBS (3 × 5 min), cells were permeabilised with Triton X‐100 (0.025% in PBS). To minimise non‐specific antibody labelling, cells were washed (3 × 5‐min PBS), incubated with 3% BSA/1% glycine (30 min, RT), washed (3 × 5‐min PBS), and blocked with 10% chick serum in PBS (30 min, RT). This was replaced with the primary monoclonal antibody against Rab5 raised in rabbit (IgG; Cell Signaling Technology Cat# 3547, RRID:AB_2300649) diluted 1:200 in 10% chick serum and incubated overnight at 4°C. Cells were then washed in PBS (3 × 5 min) and incubated with the secondary antibody, chick anti‐rabbit Alexa Fluor 488 (IgG; heavy and light chains; Cat# A‐21441; ThermoFisher; RRID:AB_2535859) 1:1000 dilution in 10% chick serum (1 hr, RT). To determine non‐specific immunofluorescent labelling, this was repeated using a secondary antibody only in the absence of the primary antibody. Cells were then washed (3 × 5‐min PBS), and nuclei were stained with 2 mg·ml^−1^ H33342 in PBS (15 min, RT) and washed (2 × 5‐min PBS) before coverslips were mounted onto slides using ProLong Diamond (Thermo Fisher Scientific) and sealed for storage at 4°C. Coverslips were imaged using a Confocal Zeiss LSM880 fitted with a 63× Pan Apochromat oil objective (1.4 numerical aperture) using a 1‐μm slice. Wavelengths were imaged in separate tracks with Rab5 immunolabelling imaged with an Argon488 laser (491‐ to 571‐nm bandpass; 3% power); VEGF_121_a‐TMR was imaged with a DPSS 561‐10 laser (571–615 nm, 3% power); and HaloTag‐Alexa Fluor 647 was imaged using a HeNe633 laser (638–747 nm; 15% power). Images were obtained at 1,024 × 1,024 pixels with eight averages and similar gains per replicate. The immuno‐related procedures used comply with the recommendations made by the *British Journal of Pharmacology*.

### 2.7 NFAT luciferase reporter gene assay

HEK293T cells stably expressing wild‐type or tyrosine phosphorylation‐deficient HaloTag‐VEGFR2, as well as NFAT‐RE‐luc2P, were grown to 70–80% confluency. Cells were seeded at 25,000 cells per well in white 96‐well plates pre‐coated with poly‐D‐lysine. Following 24 hr at 37%/5% CO_2_, cell culture media were replaced with serum‐free DMEM for another 24 hr. Cells were then stimulated with increasing concentrations of VEGF_165_a (R&D Systems) or vehicle (serum‐free DMEM/0.1% BSA). Following stimulation for 5 hr at 37%/5% CO_2_, media were replaced with 50 μl per well assay buffer and 50 μl per well ONE‐Glo Luciferase reagent (Promega Corporation, USA). Cells were incubated for 5 min to allow luciferase to react with the added reagent and for the background luminescence to subside, and then luminescence emissions were measured using a TopCount platereader (PerkinElmer, UK). Data were normalised to their respective vehicle (0%) and response of wild‐type HaloTag‐VEGFR2 to 10‐nM VEGF_165_a (100%) per experiment. Data were pooled from five independent experiments with duplicate wells.

### 2.8 Data and statistical analysis

Data were analysed using GraphPad Prism 7.02 (RRID:SCR_002798; San Diego, CA, USA). Data are presented as mean ± SEM. The data and statistical analysis comply with the recommendations of the *British Journal of Pharmacology* on experimental design and analysis in pharmacology.

Representative confocal images were processed using ImageJ (Version 1.52f; RRID:SCR_003070). For colocalisation analysis, images were corrected to the background fluorescence intensity from each experimental replicate determined using the non‐specific secondary antibody only (Rab5, 488 nm) or untransfected cells in each field of view (TMR, 546 nm; HaloTag‐VEGFR2, 647 nm). The mean background intensity was calculated for each experimental replicate (*n* = 5) and subtracted from each image for manual thresholding. To quantify colocalization, regions of interest were drawn around each cell expressing HaloTag‐VEGFR2 using the nuclei stain and phase contrast image. Following subtraction of the region outside the region of interest, colocalisation was determined using pixel‐based measures between Rab5/TMR and Rab5/HaloTag using the ImageJ plugin Coloc 2. Images with saturated pixels were excluded from analyses. Mander's overlap coefficients measure co‐occurance as the proportion of Rab5 pixels (green) overlapping with VEGF‐TMR (yellow) or HaloTag‐VEGFR2 (red). These were calculated on a per cell basis, with a total number of 64 cells (5‐min stimulation) and 68 cells (60‐min stimulation) pooled from five independent experiments.

Saturation binding curves were fitted simultaneously for total (fluorescent VEGF‐A ligand alone) and non‐specific binding (obtained in the presence of 100 nM of unlabelled VEGF‐A) using the equation:

$$\text{Total binding}=\frac{B_{\max}\times \left[B\right]}{\left[B\right]+K_{D}}+M\times \left[B\right]+C,$$

where *B*_max_ is the maximal specific binding, [*B*] the concentration of fluorescent ligand (nM), *K*_*D*_ the equilibrium dissociation constant (nM), *M* the slope of the non‐specific binding component, and *C* the *y*‐axis intercept. Background and non‐specific binding parameters were shared across all data sets.

Binding affinities (*K*_i_) of the unlabelled ligands were calculated using the Cheng–Prusoff equation:

$$K_{i}=\frac{{\mathrm{IC}}_{50}}{1+\frac{\left[L\right]}{K_{D}}},$$

where [*L*] is the concentration of fluorescent ligand used (nM). *K*_*D*_ values (nM) were derived from saturation binding curves. IC_50_ is the molar ligand concentration that will inhibit 50% of the specific binding of the fluorescent ligand concentration [*L*] and was calculated using the equation:

$$\%\text{specific VEGF binding}=\frac{100\times {\mathrm{IC}}_{50}}{\left[A\right]+{\mathrm{IC}}_{50}},$$

where [*A*] is the concentration of competing drug used.

Fluorescent ligand‐binding association kinetic data were fitted to the following mono‐exponential association function:

$$Y=Y_{\max}\cdot \left(1-e^{-k_{\mathrm{obs}}\cdot t}\right),$$

where *Y*_max_ equals the level of specific binding at infinite time, *t* is the time of incubation, and *k*_obs_ is the rate constant for the observed rate of association.

*k*_on_ and *k*_off_ values were determined by simultaneously fitting ligand‐binding association kinetic curves obtained at different fluorescent ligand concentrations (*L*) to the above equation with the following relationship between *k*_obs_ and two kinetic binding constants *k*_on_ and *k*_off_:

$$k_{\mathrm{on}=}\frac{k_{\mathrm{obs}-}k_{\mathrm{off}}}{L}.$$

Residence time was calculated as the reciprocal of *k*_off_. Kinetically determined *K*_*D*_ values were calculated from these kinetic parameters using the following equation:

$$K_{D}=\frac{k_{\mathrm{off}}}{k_{\mathrm{on}}}.$$

For VEGF_165_a‐TMR concentration–response curves for internalisation of ligand–VEGFR2 complexes, the mean granule number per cell was calculated from four images per well. Data are expressed as a percentage of the responses obtained using 100‐nM VEGF_165_a‐TMR (100%) or vehicle (0%). Data were fitted using non‐linear least squares regression using GraphPad Prism with the following equation:

$$\text{Response}=\frac{E_{\max}\times \left[B\right]}{\left[B\right]+{\mathrm{EC}}_{50}},$$

where *E*_max_ is the maximal response and EC_50_ is the concentration of agonist required to produce 50% of the maximum response.

The kinetic data for receptor internalisation were fitted to the following mono‐exponential association function:

$$Y=Y_{\max}.\left(1-e^{-k_{\mathrm{obs}}.t}\right),$$

where *Y*_max_ equals the level of receptor internalisation at infinite time, *t* is the time of incubation, and *k*_obs_ is the rate constant for the observed rate of receptor internalisation.

Statistical analyses of differences between Mander's overlap coefficients obtained at 5 and 60 min were performed using an unpaired non‐parametric Mann–Whitney test. Statistical analysis of the difference of the Mander's overlap coefficient obtained from zero were performed using a Wilcoxon signed‐rank test. Statistical analysis of differences between fitted kinetic ligand‐binding parameters, in the presence and absence of cediranib, was performed using a paired *t* test. AUC analysis (GraphPad Prism 7.02) was used to determine the effect of cediranib on NanoBRET ligand‐binding times courses (Figure 4), and the statistical significance of these changes was determined by two‐way ANOVA. Differences between kinetic binding constants in cells and membranes were performed using one‐way ANOVA with post hoc Tukey test. Statistical significance was taken as *P* < .05. A power calculation was performed to confirm sample number for statistical comparisons between binding parameters determined by NanoBRET. This was done on the basis of five separate experiments with the anticipated *SD* obtained in similar experiments and a calculation of the statistical power to detect a significant change in a particular parameter of 0.3 log units. This yielded a power of 0.99; that is, there was a 99% chance of detecting a significant change in value of 0.3 log units.

### 2.9 Nomenclature of targets and ligands

Key protein targets and ligands in this article are hyperlinked to corresponding entries in http://www.guidetopharmacology.org, the common portal for data from the IUPHAR/BPS Guide to PHARMACOLOGY (Harding et al., 2018), and are permanently archived in the Concise Guide to PHARMACOLOGY 2017/18 (Alexander, Fabbro et al., 2017; Alexander, Kelly et al., 2017).

## 3 RESULTS

### 3.1 Binding kinetics of VEGF_165_b and VEGF_121_a in intact living HEK293 cells

**Figure 1:**
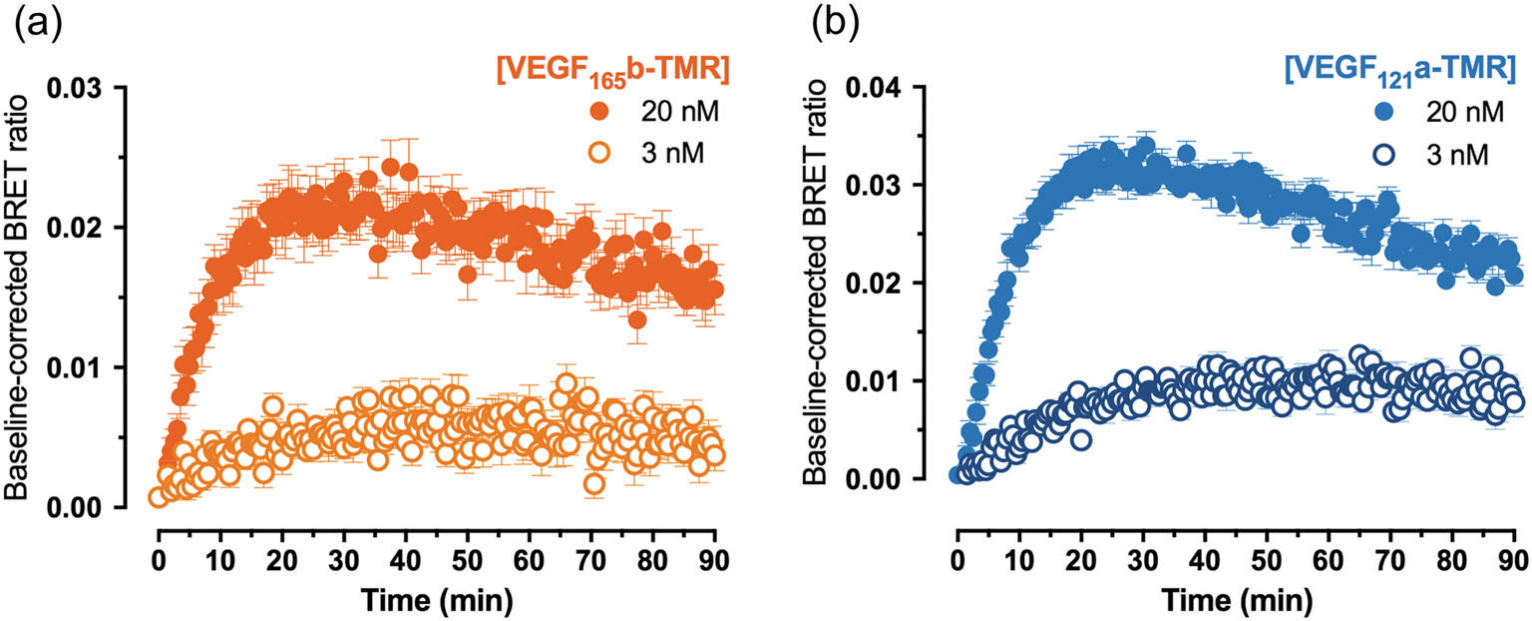
Long‐term NanoBRET binding profiles of fluorescent VEGF‐A variants at NanoLuc‐tagged VEGFR2 expressed in intact living cells. (a) Time course of VEGF_165_b‐TMR and (b) VEGF_121_a‐TMR binding to full‐length N‐terminal NanoLuc‐VEGFR2 stably expressed in HEK293T cells. Following 5 min pretreatment with NanoLuc substrate furimazine, ligand was added (x = 0) at both a saturating (20 nM) concentration of fluorescent ligand and a concentration that was approximately equal to its K_D_ value (3 nM). BRET ratios were calculated every 30 s at 37°C using the PHERAstar FS platereader. Data were baseline corrected to vehicle to adjust for background emissions. Data are shown as mean ± SEM from five independent experiments with duplicate wells

Initial kinetic studies with VEGF_165_b‐TMR and VEGF_121_a‐TMR recapitulated the previous findings obtained with VEGF_165_a‐TMR in intact living cells (Kilpatrick et al., 2017). Thus, with concentrations chosen to be close to their respective *K*_*D*_ values (3 nM), the specific binding of each ligand increased rapidly and was then relatively well maintained over the course of the experiment (Figure 1a,b). The specific binding observed with 20‐nM VEGF_165_b‐TMR or VEGF_121_a‐TMR, however, peaked at *~*20 min and then declined substantially towards baseline over the next 70 min (Figure 1a,b).

### 3.2 Agonist‐mediated internalisation of VEGFR2 in HEK293T cells

**Figure 2:**
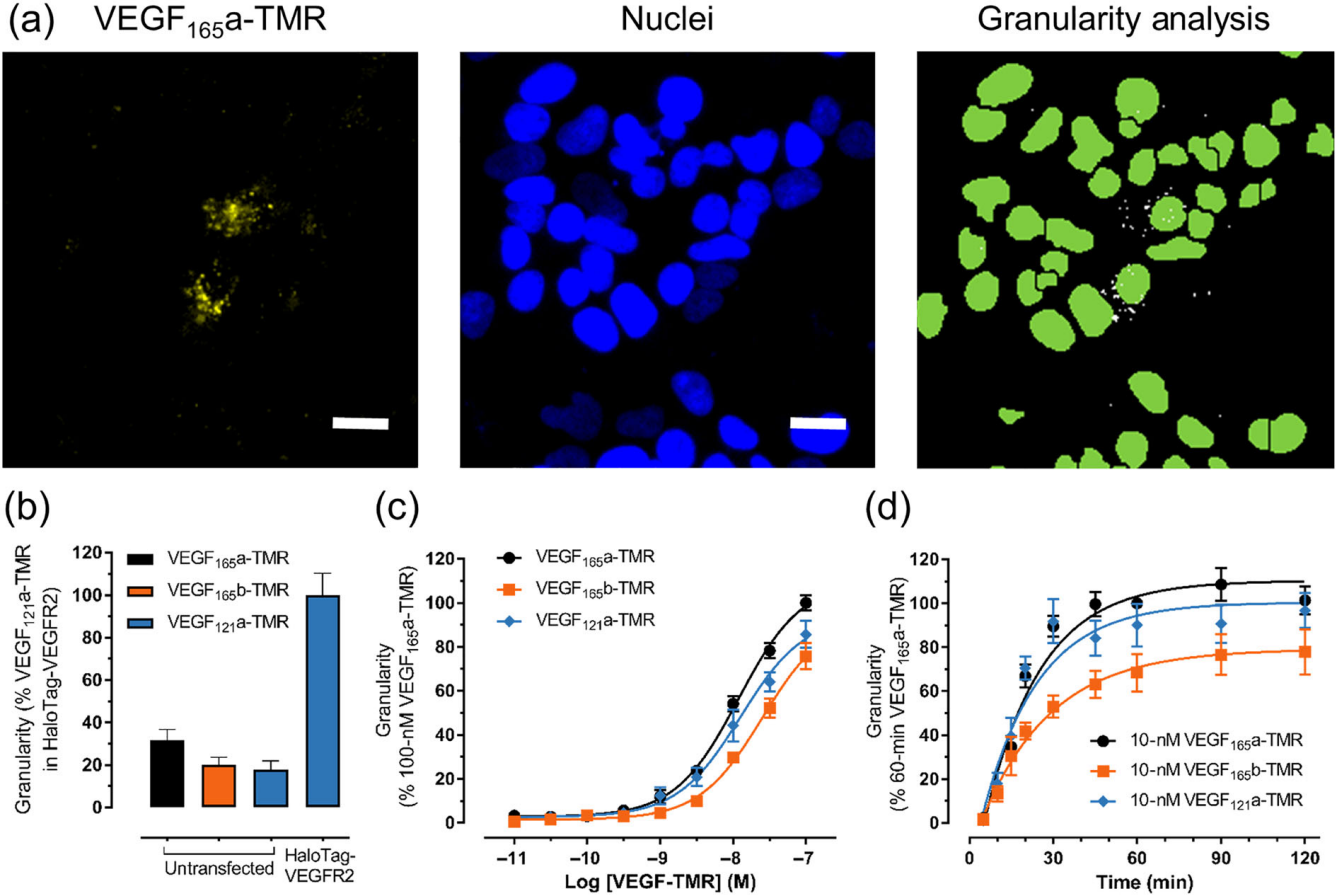
Quantifying endocytosis of VEGF_165_a‐TMR, VEGF_165_b‐TMR, and VEGF_121_a‐TMR ligand–receptor complexes using high‐content imaging. (a) HEK293T cells expressing NanoLuc‐VEGFR2 were stimulated with 10‐nM fluorescent VEGF_165_a‐TMR (60 min, 37°C). Cells were fixed and incubated with nuclear stain (H33342). The following day, cells were imaged with an ImageXpress Micro widefield platereader (20× ELWD objective; four sites per well) using filter settings for TRITC (left; VEGF_165_a‐TMR) and DAPI (middle; nuclei). Images were analysed using a granularity algorithm (Molecular Devices) whereby nuclei stained with H33342 (blue, middle panel) were identified based upon their size (5‐ to 25‐μm diameter) and pixel depth in grey levels. A nuclear mask defining cell nuclei was then placed over the nuclei within the acquired image (right panel; green spots), forming the basis for automated image segmentation. Fluorescent granules detected in the TRITC channel (right; white spots) were defined based upon size (2‐ to 15‐μm diameter) and pixel depth in grey levels. Granules were then assigned to specific nuclei based upon proximity using the aforementioned segmented image. Scale bars represent 20 μm. (b) To confirm receptor specificity, non‐transfected wild‐type HEK293T cells or stably expressing HaloTag‐VEGFR2 cells were stimulated with 10‐nM VEGF_165_a‐TMR (n = 5), VEGF_165_b‐TMR (n = 5), or VEGF_121_a‐TMR (n = 5) for 60 min (37°C). Cells were fixed, stained, and imaged, as in (a). Data were normalised to mean vehicle (0%) or VEGF_121_a‐TMR stimulation (100%) in HaloTag‐VEGFR2 cells per experiment. (c) Cells were stimulated with increasing concentrations of fluorescent VEGF‐A variants (60 min, 37°C). Data were normalised to mean vehicle (0%) and 100‐nM VEGF_165_a‐TMR‐stimulated response (100%) per experiment. Cells were fixed, stained, and imaged as above. (d) Timecourse of internalisation of VEGF‐TMR complexes, whereby NanoLuc‐VEGFR2 cells were stimulated with 10‐nM VEGF_165_a‐TMR, VEGF_165_b‐TMR, or VEGF_121_a‐TMR for 0–120 min at 37°C. Cells were fixed, stained, and imaged as above and then normalised to vehicle (0%) and 60‐min VEGF_165_a‐TMR stimulation (100%) per experiment. Data from (b–d) are expressed as mean ± SEM and pooled from five independent experiments unless stated otherwise with duplicate wells imaged at four sites per well

We have previously suggested that the fall in the VEGFR2‐associated NanoBRET signal observed with VEGF_165_a‐TMR over longer incubations periods is a consequence of VEGFR2 internalisation and the dissociation of VEGF_165_a‐TMR from its receptor within intracellular endosomes (Kilpatrick et al., 2017). To look into the timecourse and potency of receptor internalisation by each fluorescent VEGF‐A isoform, we investigated receptor endocytosis by monitoring the appearance of fluorescent ligand‐associated receptors in intracellular endosomes using unbiased, automated quantitative high‐content imaging. A granularity algorithm was used to detect the presence of fluorescent VEGF‐A isoforms within granules of a particular size (i.e. between 2 and 15 μm; Figure 2a). We have previously shown that this analysis correlates with the endocytosis of tagged receptors (Kilpatrick et al., 2017). Minimal VEGF‐TMR containing granules were detected in untransfected HEK293T cells compared with cells stably expressing HaloTag‐VEGFR2 (Figure 2b). All three fluorescent VEGF‐A isoforms stimulated a potent internalisation of VEGFR2 (Figure 2c and Table 1). In the presence of 10 nM of VEGF_165_a‐TMR, VEGF_165_b‐TMR, or VEGF_121_a‐TMR, the appearance of ligand‐bound VEGFR2 in endosomes was rapid with a *t*_1/2_ of between 15 and 20 min (Figure 2d and Table 1).

**Table 1:** Concentration–response parameters and k_obs_ values for fluorescent VEGF‐A‐induced VEGFR2 internalisation in HEK293T cells expressing NanoLuc‐VEGFR2 derived from experiments similar to those described in Figure 2

Ligand	pEC_50_	Observed rate constant (k_obs_, min^−1^)	t_1/2_ (min)
VEGF_165_a‐TMR	7.95 ± 0.09	0.04 ± 0.01	17.5 ± 1.87
VEGF_165_b‐TMR	7.54 ± 0.08	0.04 ± 0.01	19.8 ± 3.53
VEGF_121_a‐TMR	7.81 ± 0.12	0.05 ± 0.01	14.8 ± 2.11

*Note*. Data are mean ± SEM from five separate experiments.

### 3.3 Presence of VEGF‐TMR and VEGFR2 in Rab5+ endosomes

**Figure 3:**
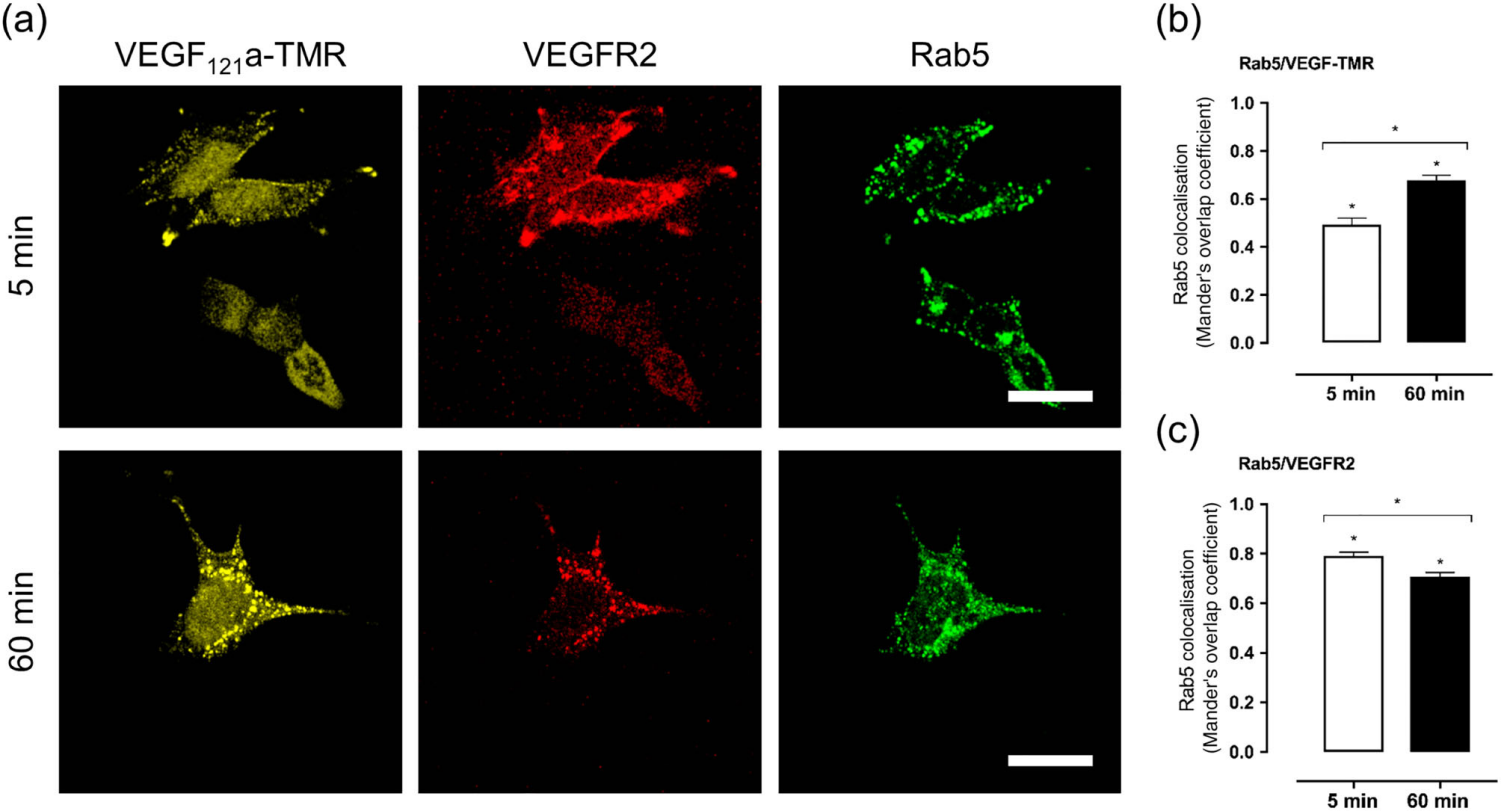
Localisation of VEGFR2 and VEGF_121_a‐TMR in Rab5+ endosomes. (a) HEK293T cells expressing HaloTag‐VEGFR2 were labelled with membrane‐impermeant HaloTag‐Alexa Fluor 647 (red) and stimulated with 10‐nM VEGF_121_a‐TMR (yellow) for 5 or 60 min at 37°C. Cells were fixed and permeabilised using Triton X‐100 (0.025% in PBS), and endosomal compartments were labelled with a primary monoclonal Rab5 antibody and secondary chick anti‐rabbit Alexa Fluor 488 antibody (green). Coverslips were imaged using a Zeiss LSM880 (63× oil objective), with representative images obtained on the same day. Scale bar represents 20 μm. (b, c) Colocalisation parameters were quantified based on regions of interest drawn around cells positive for HaloTag‐VEGFR2 expression using ImageJ software for Coloc 2 analysis. The Mander's overlap coefficient represents the proportion of Rab5 (green) colocalised with (b) ligand (VEGF_121_a‐TMR; yellow) or (c) receptor (HaloTag‐VEGFR2; red), following stimulation for 5 or 60 min. All coefficient values were pooled from five independent experiments, with a total of 68 cells (5‐min stimulation) or 64 cells (60‐min stimulation). Coefficients obtained at 5 and 60 min were compared using an unpaired Mann–Whitney test (*P < .05; n = 68 or n = 64). Coefficients were tested for a significant difference (*P < .05) from zero using a Wilcoxon signed‐rank test

Immunofluorescent labelling of Rab5 in fixed cells was used to confirm whether VEGF‐TMR and VEGFR2 complexes were localised in early Rab5‐positive endosomes, using confocal microscopy for enhanced axial resolution (*z*) compared with high‐content widefield imaging in Figure 2. As a representative VEGF‐TMR ligand, 10‐nM VEGF_121_a‐TMR was significantly colocalised with Rab5 (*P* < .05; Wilcoxon signed‐rank test; Figure 3a,b) at both intracellular sites and regions of the plasma membrane by 5 min, whereas it was largely intracellular by 60 min (Figure 3a,b). This was accompanied by increased colocalisation with Rab5 at 60 min (Figure 3b; *P* < .05; Mann–Whitney test, *n* = 64 cells and *n* = 68 cells for 5‐ and 60‐min stimulation, respectively). HaloTag‐VEGFR2 labelled with a membrane‐impermeable dye was significantly (*P* < .05) colocalised with Rab5 at 5 min (Figure 3a,c). At 60 min, there was a small decrease in colocalisation of Rab5 with HaloTag‐VEGFR2 compared with 5 min (Figure 3c; Mann–Whitney test, *P* < .05; *n* = 64 for 5 min and *n* = 68 for 60 min), which is consistent with some recycling of VEGFR2 back to the plasma membrane previously observed by Kilpatrick et al. (2017).

### 3.4 Influence of VEGFR2 phosphorylation on ligand binding in intact cells

**Figure 4:**
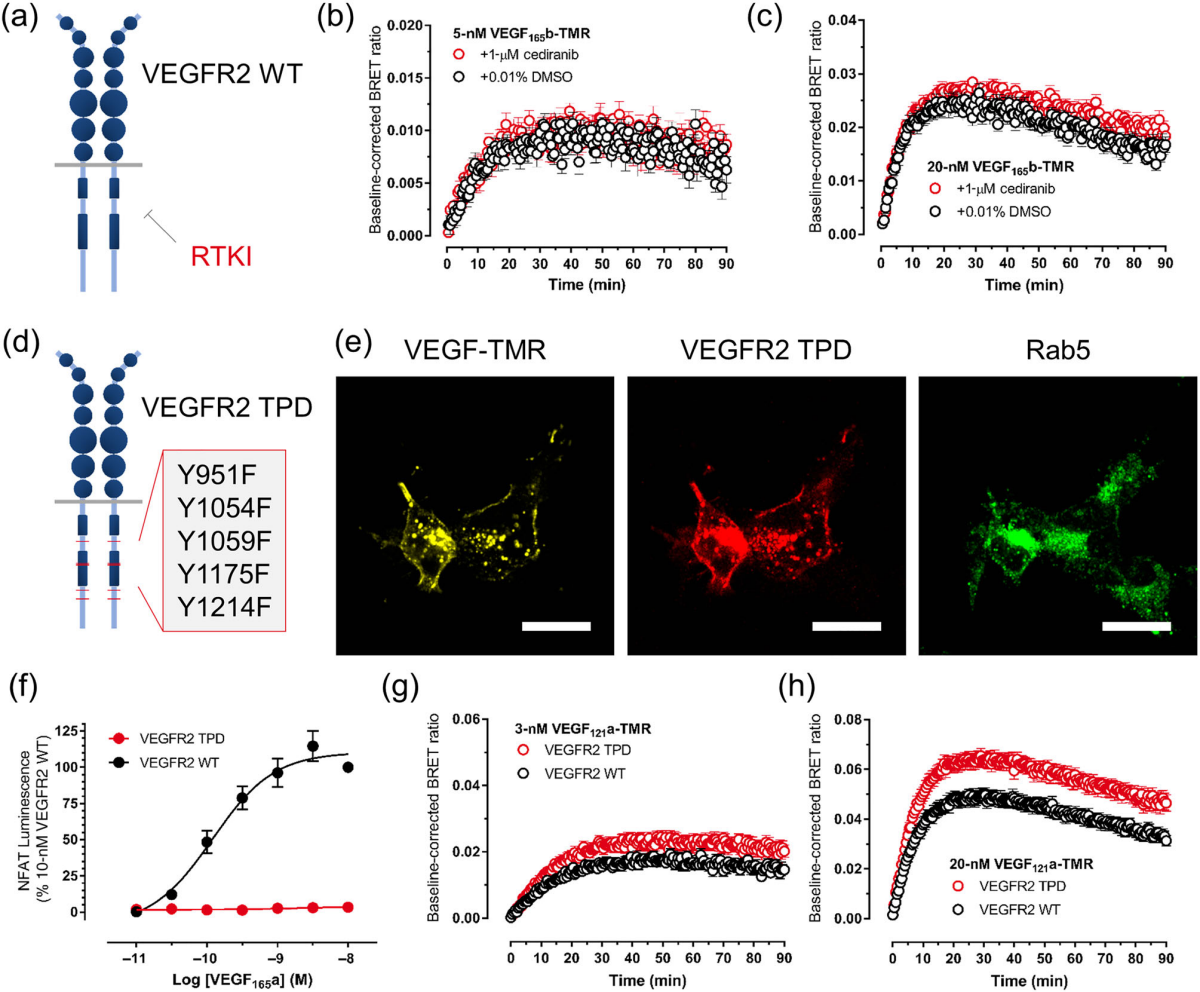
VEGFR2 phosphorylation is not required for the decline in NanoBRET signal. (a) Schematic representing the mechanism of cediranib at the intracellular ATP‐binding site of VEGFR2. (b, c) HEK293T cells stably expressing NanoLuc‐VEGFR2 were pretreated with 0.01% DMSO (control) or 1‐μM cediranib (30 min, 37°C). After the addition of furimazine for 5 min, the real‐time binding of (b) 5‐ or (c) 20‐nM VEGF_165_b‐TMR was monitored every 30 s for 90 min at 37°C. Data are shown as mean ± SEM from five independent experiments with duplicate wells. AUC analysis provided the following areas (mean ± SEM): (b) 0.71 ± 0.01 and 0.77 ± 0.01* and (c) 1.80 ± 0.02 and 2.04 ± 0.02* for control and cediranib‐treated cells respectively. *P < .05 significant difference between control and cediranib‐treated cells (two‐way ANOVA of every time point). (d) Schematic of tyrosine mutations in the tyrosine phosphorylation‐deficient VEGFR2‐TPD. (e) HEK293T cells stably expressing HaloTag‐VEGFR2‐TPD were labelled with membrane‐impermeant HaloTag‐Alexa Fluor AF647 and stimulated with 10‐nM VEGF_121_a‐TMR (60 min, 37°C). Following cell fixation and permeabilization, Rab5‐positive endosomes were labelled with a monoclonal rabbit antibody and secondary chick anti‐rabbit Alexa Fluor 488. Cells were imaged using a Zeiss LSM880F Confocal (63× oil objective) with representative images from five independent experiments. Scale bars show 20 μm. (f) NFAT luciferase production in response to 5‐hr stimulation with increasing concentrations of VEGF_165_a. Stable cell lines were then used for Rab5+ confocal imaging figures to confirm cell surface expression. Data are shown as mean ± SEM from five independent experiments in duplicate wells, expressed as a percentage of response per experiment to 10‐nM VEGF_165_a at wild‐type HaloTag‐VEGFR2 (100%) or respective vehicle (0%). (g, h) Time course of VEGF_121_a‐TMR binding to tyrosine phosphorylation‐deficient NanoLuc‐VEGFR2 expressed transiently in HEK293T cells. Cells were pretreated with furimazine for 5 min, and then (g) 3‐ or (h) 20‐nM ligand was added (x = 0). BRET ratios were monitored every 30 s at 37°C and baseline corrected to vehicle. Data are expressed as mean ± SEM from eight independent experiments with duplicate wells. AUC analysis provided the following areas (mean ± SEM): (g) 2.61 ± 0.06 and 3.54 ± 0.07* and (h) 7.28 ± 0.10 and 9.79 ± 0.11*, for wild‐type VEGFR2 and VEGFR2‐TPD cells respectively. *P < .05 significant difference between VEGFR2 and VEGFR2‐TPD cells (two‐way ANOVA of every time point)

Addition of the RTKI cediranib (1 μM), pre‐incubated for 30 min, produced a significant elevation in the NanoBRET signal at both 5‐ and 20‐nM VEGF_165_b‐TMR relative to the DMSO control (Figure 4a–c; *P* < .05, two‐way ANOVA of every time point, *n* = 5). This elevation was similar to that reported previously with an endpoint NanoBRET binding timecourse for VEGF_165_a‐TMR (Kilpatrick et al., 2017). However, as also noted by Kilpatrick et al. (2017), the binding profile of 20‐nM VEGF_165_b‐TMR continued to decline in the presence of cediranib following the peak at 20 min when monitored continuously using real‐time NanoBRET measurements (Figure 4c).

To explore the influence of receptor phosphorylation further, we generated a tyrosine phosphorylation‐deficient VEGFR2 (VEGFR2‐TPD) where key intracellular phosphotyrosine residues were mutated to phenylalanine (Y951F, Y1054F, Y1059F, Y1175F, and Y1214F; Figure 4d). Receptors were labelled with membrane‐impermeant HaloTag‐Alexa Fluor 647, and this confirmed the plasma membrane expression of this VEGFR2 variant (Figure 4e). However, VEGF_121_a‐TMR and VEGFR2‐TPD were also localised to intracellular Rab5+ sites following a 60‐min incubation. Using the same cell line as those for imaging experiments, the tyrosine phosphorylation‐deficient VEGFR2 was unable to signal through NFAT in response to increasing concentrations of VEGF_165_a in comparison with wild‐type VEGFR2 (pEC_50_ = 9.92 ± 0.12; Figure 4f). In intact cells, there was a significant elevation (*P* < .05) in NanoBRET signal with both 3‐ and 20‐nM VEGF_121_a‐TMR (Figure 4g,h; *n* = 8). It was notable that there was also a decline in the NanoBRET signal in VEGFR2‐TPD after the initial peak at 20 min. Both wild‐type and tyrosine phosphorylation‐deficient NanoLuc‐VEGFR2 receptors were expressed at the same level, as determined by the NanoLuc luminescence emissions (data not shown).

### 3.5 NanoBRET ligand binding in membrane preparations

**Figure 5:**
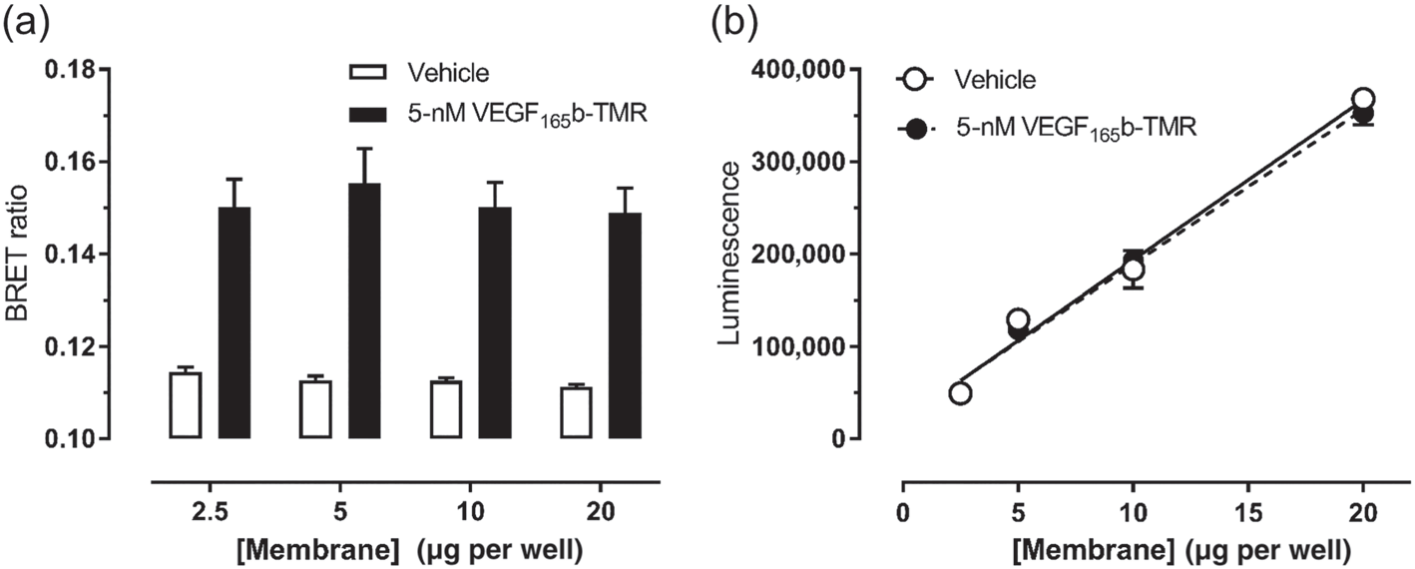
Influence of membrane concentration on ligand binding between NanoLuc‐tagged receptors and fluorescent VEGF_165_b‐TMR detected using NanoBRET. (a) Increasing concentrations of membranes prepared from HEK293T cells expressing NanoLuc‐VEGFR2 were incubated with vehicle or 5‐nM VEGF_165_b‐TMR. Following 60‐min incubation at 37°C, BRET ratios were calculated and expressed as mean ± SEM. Data were pooled from five independent experiments using separate membrane preparations per experiment. (b) Variation in NanoLuc luminescence detected at different membrane concentrations in a single representative experiment with duplicate wells

To determine the influence of membrane concentrations on NanoBRET ligand binding in membranes prepared from NanoLuc‐VEGFR2‐expressing HEK293T cells, we initially investigated the binding of 5‐nM VEGF_165_b‐TMR to VEGFR2 over a range of membrane concentrations (2.5‐ to 20‐μg protein per well). NanoBRET binding was well maintained across the full range of protein concentrations (Figure 5a). Membrane concentration was also linearly related to NanoLuc luminescence (Figure 5b).

**Figure 6:**
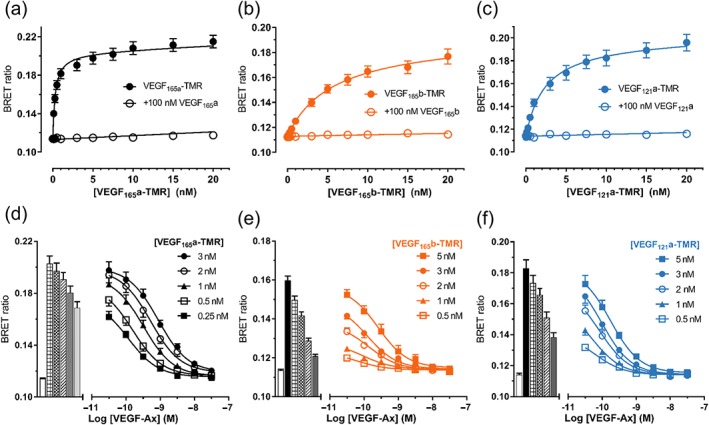
Binding characteristics of fluorescent VEGF‐A isoforms at NanoLuc‐tagged receptors in membrane preparations. Membrane preparations from HEK293T cells expressing wild‐type NanoLuc‐VEGFR2 (5 μg per well) were incubated with increasing concentrations of (a) VEGF_165_a‐TMR, (b) VEGF_165_b‐TMR, and (c) VEGF_121_a‐TMR (60 min, 37°C). Non‐specific binding was determined using 100‐nM unlabelled VEGF_xxx_x counterpart (R&D Systems) added simultaneously with fluorescent ligand. Raw BRET ratios are expressed as mean ± SEM from five independent experiments. Displacement of unlabelled VEGF‐Ax by (d) VEGF_165_a‐TMR (n = 5), (e) VEGF_165_b‐TMR (n = 6), or (f) VEGF_121_a‐TMR (n = 5) in NanoLuc‐VEGFR2 membrane preparations. Increasing concentrations of unlabelled ligand were added simultaneously with five fixed concentrations of fluorescent ligand and incubated for 60 min at 37°C. Values are mean ± SEM from five to six independent experiments with duplicate wells. Bars show the binding obtained with vehicle (white) or fluorescent ligand alone (left to right; highest to lowest concentration of each fluorescent ligand used)

The binding of different concentrations of fluorescent VEGF‐A isoforms monitored by NanoBRET showed clear saturable specific binding in each case. The *K*_*D*_ values obtained in membrane preparations from these equilibrium binding experiments were slightly more potent (0.31 ± 0.06, 5.55 ± 0.47, and 2.58 ± 0.34 nM for VEGF_165_a‐TMR, VEGF_165_b‐TMR, and VEGF_121_a‐TMR, respectively; *n* = 5 in each case) than those similarly determined in intact cells (2.03, 9.53, and 5.54 nM; Peach, Kilpatrick, et al., 2018). However, non‐specific binding was low in all cases (Figure 6a–c). Competition binding experiments were also conducted for each fluorescent VEGF‐A isoform with VEGF‐Ax yielding similar pK_i_ values for the non‐fluorescent ligand (10.18 ± 0.04 [*n* = 5], 9.96 ± 0.2 [*n* = 6], and 10.34 ± 0.08 [*n* = 5]; using VEGF_165_a‐TMR, VEGF_165_b‐TMR, and VEGF_121_a‐TMR as the fluorescent probe, respectively; Figure 6d–f).

### 3.6 Kinetics of the binding of fluorescent VEGF‐A isoforms to VEGFR2 in membrane preparations

**Figure 7:**
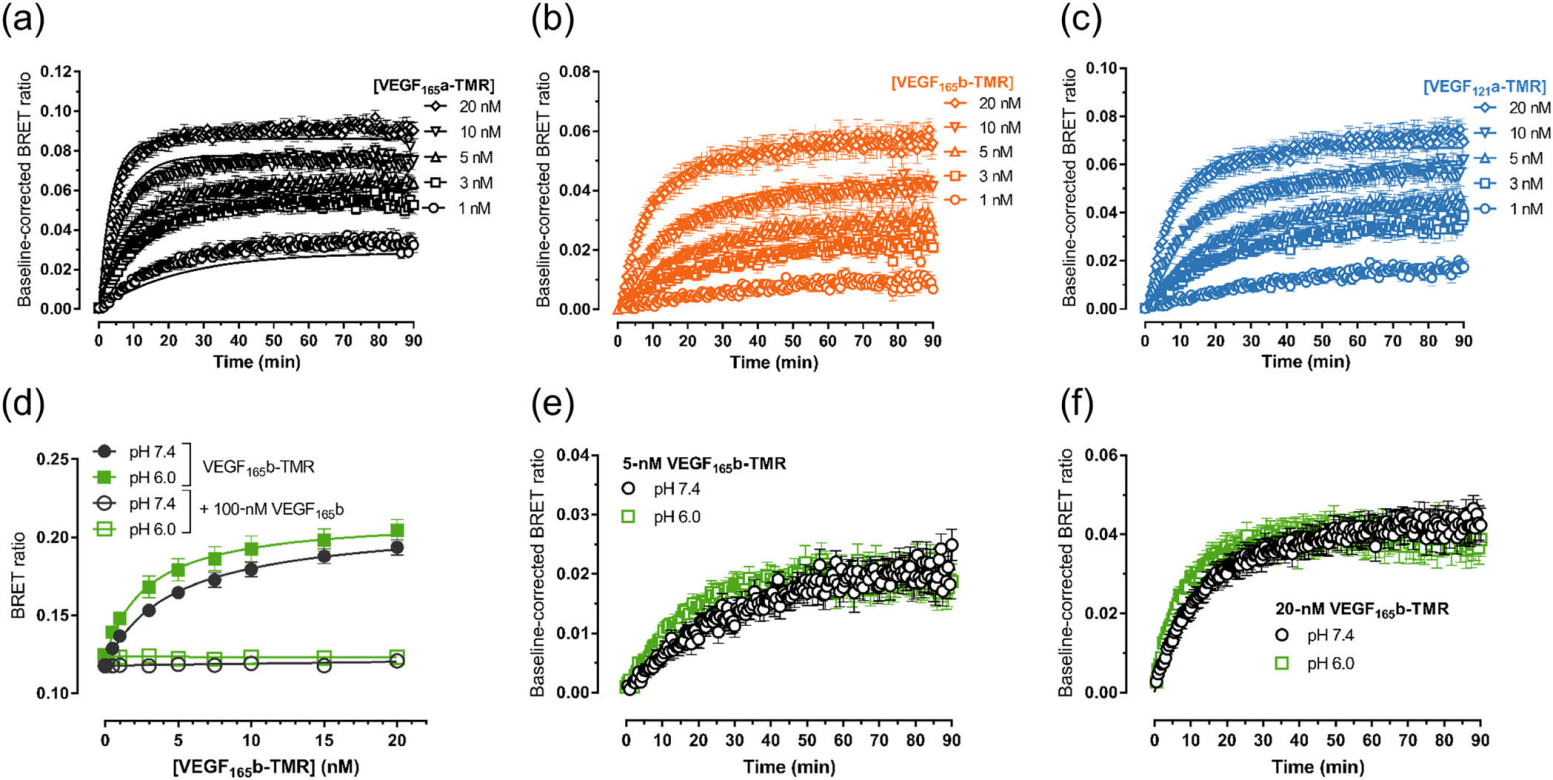
Binding kinetics of VEGF_165_a‐TMR, VEGF_165_b‐TMR, and VEGF_121_a‐TMR in membrane preparations quantified using NanoBRET. Membranes (10 μg per well) prepared from NanoLuc‐VEGFR2‐expressing HEK293T cells were pretreated with furimazine to equilibrate for 5 min before addition of five concentrations of (a) VEGF_165_a‐TMR, (b) VEGF_165_b‐TMR, and (c) VEGF_121_a‐TMR (1–20 nM). Measurements were taken every 30 s for 90 min at 37°C and baseline corrected to vehicle. Data represent mean ± SEM from five independent experiments with duplicate wells. Individual curves were fit with a simple exponential association model. (d) Saturation binding of VEGF_165_b‐TMR at NanoLuc‐VEGFR2 in membrane preparations (5 μg per well), comparing assay buffer at pH 7.4 and 6.0 (HEPES buffered saline solution containing 0.1% BSA). Membranes were incubated with increasing concentrations of VEGF_165_b‐TMR for 60 min at 37°C, in the presence and absence of 100‐nM VEGF_165_b (R&D Systems) to determine non‐specific binding. Raw BRET ratios are expressed as mean ± SEM from six independent experiments with duplicate wells. Kinetics of (e) 5‐ and (f) 20‐nM VEGF_165_b‐TMR at NanoLuc‐VEGFR2 in membrane preparations (10 μg per well), comparing assay buffer at pH 7.4 and pH 6.0 (HEPES buffered saline solution/0.1% BSA). The binding profile for single VEGF_165_b‐TMR concentrations are shown for clarity; however, experiments were performed with four concentrations (3–20 nM) to fit a global kinetic association model, as above. Data represent mean ± SEM from six independent experiments with duplicate wells

To determine the kinetic constants (*k*_on_ and *k*_off_) for the binding of fluorescent VEGF‐A isoforms to VEGFR2, we investigated the timecourse of the binding of five concentrations of VEGF_165_a‐TMR, VEGF_165_b‐TMR, and VEGF_121_a‐TMR at NanoLuc‐VEGFR2 in membrane preparations (1–20 nM; Figure 7a–c). Binding of all three fluorescent VEGF‐A isoforms produced classic ligand‐binding association curves that, in contrast to the studies in intact cells (Figure 1), were maintained for the duration of the experiment (90 min; Figure 7a–c). Kinetic *k*_on_ and *k*_off_ values determined from these experiments in Table 2 were similar to those previously reported for these three ligands in intact cells, where the kinetic analysis was limited to the first 20 min of agonist stimulation (Peach, Kilpatrick, et al., 2018).

**Table 2:** Kinetic binding parameters of ligand binding to NanoLuc‐VEGFR2 in isolated membranes and intact cells

Ligand	HEK293T membrane preparations				Intact HEK293T cells			
	k_on_ (min^−1^·M^−1^)	k_off_ (min^−1^)	Kinetic K_D_ (nM)	Residence time (min)	k_on_ (min^−1^·M^−1^)	k_off_ (min^‐1)^	Kinetic K_D_ (nM)	Residence time (min)
VEGF_165_a‐TMR	1.27 × 10^7^ ± 0.24 × 10^7^	0.030 ± 0.001	2.21 ± 0.40	33.3	1.54 × 10^7^	0.06	6.64	16.7
VEGF_165_b‐TMR	3.69 × 10^6^ ± 0.67 × 10^6^	0.029 ± 0.002	9.21 ± 2.53	34.5	7.29 × 10^6^	0.06	11.3	16.7
VEGF_121_a‐TMR	5.13 × 10^6^ ± 0.48 × 10^6^	0.019 ± 0.002	3.85 ± 0.61	52.6	8.51 × 10^6^	0.05	5.54	20.0

*Note*. Values in membrane preparations are mean ± SEM (*n* = 5) derived from experiments shown in Figure 7 using five concentrations of fluorescent ligand. Values in intact cells are taken from Peach, Kilpatrick, et al. (2018). *K*_*D*_ values shown are the kinetically derived values (*k*_off_/*k*_on_).

Membrane kinetic experiments were also repeated in the presence of the RTKI cediranib (Table 3). There was a significant decrease in *k*_off_ in membrane preparations when compared with cells (Table 3), but the *k*_off_ values were not altered by cediranib treatment. There was a small significant decrease in the *k*_on_ value for VEGF_165_b‐TMR obtained in intact cells in the presence of cediranib compared with the DMSO control. The value obtained in the presence of cediranib was also similar to the *k*_on_ values obtained in membranes (Table 3). To quantify the kinetics of binding at the tyrosine phosphorylation‐deficient NanoLuc‐VEGFR2 using membrane preparations, a stable cell line was generated. Kinetic parameters of VEGF_121_a‐TMR in VEGFR2‐TPD membranes (Table 3) were similar to control VEGF_121_a‐TMR kinetic parameters derived in wild‐type VEGFR2 membranes (Tables 2 and 3).

**Table 3:** Effect of cediranib and tyrosine phosphorylation deficiency on the binding kinetics of VEGF_165_b‐TMR to NanoLuc‐VEGFR2

**Cells or membranes**	Ligand	Drug	k_on_ (min^−1^·M^−1^)	k_off_ (min^−1^)	Residence time (min)	Kinetic K_D_ (nM)
VEGFR2 WT cells^a^	VEGF_165_b‐TMR (5)	0.01% DMSO	4.26 × 10^6^ ± 0.50 × 10^6^	0.081 ± 0.009	12.3	19.8 ± 2.96
		1‐μM cediranib	2.63 × 10^6^ ± 0.51 × 10^6^ *	0.091 ± 0.015	11.0	43.6 ± 14.5
VEGFR2 WT membranes^b^	VEGF_165_b‐TMR (5)	0.01% DMSO	2.69 × 10^6^ ± 0.30 × 10^6^ *	0.023 ± 0.003*	43.5	9.68 ± 2.70
		1‐μM cediranib	2.74 × 10^6^ ± 0.28 × 10^6^ *	0.021 ± 0.002*	47.6	8.00 ± 1.52
VEGFR2‐TPD membranes^b^	VEGF_121_a‐TMR (5)	None	6.21 × 10^6^ ± 1.61 × 10^6^	0.037 ± 0.006	27.0	6.85 ± 1.81
VEGFR2 WT membranes^b^	VEGF_165_b‐TMR (6)	pH 7.4	2.34 × 10^6^ ± 0.19 × 10^6^	0.023 ± 0.002	43.5	10.2 ± 1.20
		pH 6.0	4.39 × 10^6^ ± 0.39 × 10^6^ #	0.059 ± 0.019#	17.0	13.3 ± 3.55

*Note*. Values shown are mean ± SEM, with the number of independent experiments in parentheses. Data were taken from kinetic binding experiments at wild‐type NanoLuc‐VEGFR2 (VEGFR2 WT) in cells or membranes, or tyrosine phosphorylation‐deficient NanoLuc‐VEGFR2 (VEGFR2‐TPD) membranes.

a Kinetic parameters were determined by a global association kinetic fit to four concentrations of fluorescent ligand from initial 20 min in intact cells.

b Kinetic parameters were determined by a global association kinetic fit to four concentrations of fluorescent ligand from 90 min in membrane preparations.

* *P* < .05 for comparison with the *k*_on_ and *k*_off_ values determined in 0.01% DMSO in VEGFR2 WT cells (one‐way ANOVA with post hoc Tukey test) compared with DMSO control in VEGFR2 WT cells.

# *P* < .05; Wilcoxon matched‐pairs signed‐rank test (pH 7.4 vs. pH 6.0). Matched analysis of paired kinetic constants for 0.01% DMSO versus cediranib in either cells or membranes was not significant.

Taking advantage of our binding assay in membrane preparations, we investigated the effect of an acidic pH on ligand–receptor binding to reflect early endosomes (pH 6.0) compared with physiological extracellular pH (7.4, as above). Using VEGF_165_b‐TMR as a representative ligand at membranes expressing wild‐type NanoLuc‐VEGFR2, there was comparable saturable binding with low non‐specific binding (Figure 7d). Equilibrium binding constants for VEGF_165_b‐TMR were similar at neutral and acidic pH (pH 7.4, *K*_*D*_ = 5.29 ± 1.12 nM, and pH 6.0, *K*_*D*_ = 4.43 ± 1.25 nM; *n* = 6 in both experiments). Kinetic experiments showed similar association binding of VEGF_165_b‐TMR at both pH conditions (Figure 7e,f; *n* = 6). Kinetic parameters derived using four concentrations of VEGF_165_b‐TMR show kinetics at an acidic pH had faster association and dissociation rate constants (Table 3). The *K*_*D*_ affinity estimated from kinetic parameters (*k*_off_/*k*_on_) also yielded a similar binding affinity at both pH conditions, despite the difference in residence times.

## 4 DISCUSSION

Initial kinetic studies of the ligand binding of VEGF_165_b‐TMR and VEGF_121_a‐TMR, in intact HEK293T cells, recapitulated previous findings obtained with VEGF_165_a‐TMR in intact living cells (Kilpatrick et al., 2017). We have previously reported all three fluorescent VEGF‐A isoforms bind with nanomolar affinity to VEGFR2 (Kilpatrick et al., 2017; Peach, Kilpatrick, et al., 2018). Thus, at concentrations close to their respective *K*_*D*_ values, the specific binding of each ligand (monitored by NanoBRET) increased rapidly and was then relatively well maintained over the course of the experiment. In contrast, specific binding observed with higher concentrations (e.g., 20 nM) of VEGF_165_b‐TMR or VEGF_121_a‐TMR peaked at *~*20 min and then declined substantially towards baseline over the next 70 min. Previous studies with VEGF_165_a‐TMR alone suggested that this was a consequence of VEGFR2 endocytosis and the dissociation of the fluorescent ligand from the receptor within intracellular endosomes, followed by subsequent recycling of ligand‐free VEGFR2 back to the plasma membrane (Kilpatrick et al., 2017). Published studies in primary endothelial cells using immunofluorescence antibody labelling or biochemical techniques agreed that VEGFR2 internalised within 30–60 min of VEGF‐A stimulation (Bruns et al., 2010; Ewan et al., 2006; Jopling et al., 2009).

To determine the agonist potency (EC_50_ values) and kinetic profile of agonist‐induced VEGFR2 endocytosis, we monitored the appearance of fluorescent ligand‐associated receptors in intracellular endosomes using high‐content quantitative imaging. This approach was able to quantify the internalisation of VEGF‐A ligand isoforms independently of the known constitutive endocytosis of VEGFR2 (Basagiannis et al., 2016; Ewan et al., 2006; Jopling et al., 2009; Jopling et al., 2011). All three fluorescent VEGF‐A isoforms stimulated comparable internalisation of VEGFR2 (EC_50_ = 12–30 nM), with 10 nM of each fluorescent ligand eliciting a rapid appearance of ligand‐bound VEGFR2 in endosomes (*t*_1/2_ of between 15 and 20 min). This contrasted with previous findings in HUVEC that VEGF_121_a was less able to induce VEGFR2 endocytosis compared with VEGF_165_a (Fearnley et al., 2016). However, it is notable that the HEK293T cells used in the present study have minimal expression of the co‐receptor neuropilin 1 (Peach, Kilpatrick, et al., 2018), whereas human umbilical vein endothelial cells also express the co‐receptor neuropilin 1. Neuropilin 1 does not interact with VEGF_121_a (Peach, Kilpatrick, et al., 2018) but has been reported to modulate VEGFR2 trafficking (Ballmer‐Hofer, Andersson, Ratcliffe, & Berger, 2011). We were, however, able to confirm using confocal microscopy that both VEGF_121_a‐TMR and VEGFR2 were colocalised with Rab5 in intracellular endosomes following addition of the fluorescent ligand.

The data obtained from the internalisation assays also indicate that significant internalisation only occurs at the highest concentrations of fluorescent VEGF‐A isoforms used (EC_50_ = 12, 14, and 30 nM for VEGF_165_a‐TMR, VEGF_121_a‐TMR, and VEGF_165_b‐TMR, respectively). Interestingly, these values are greater than the corresponding kinetically derived *K*_*D*_ ligand‐binding values determined in membrane preparations (2.4, 3.9, and 9.2 nM). This might suggest that the binding affinity of VEGFR2 for VEGF‐A isoforms is lower after receptor activation as a result of autophosphorylation and engagement with signalling proteins involved in endocytosis. To investigate the impact of receptor TK activation on this process, we have investigated the effect of pretreating cells with the RTKI cediranib (Carter et al., 2015). Cediranib (1 μM) produced no significant effect on the kinetic rate constants (*k*_on_ and *k*_off_) or *K*_*D*_ value of VEGF_165_b‐TMR determined from binding to VEGFR2 in HEK293T cell membranes. In intact cells, however, cediranib produced a small elevation in the NanoBRET signal similar to that reported previously for VEGF_165_a‐TMR (Kilpatrick et al., 2017). This was accompanied by a small decrease in *k*_on_ but no significant difference in *k*_off_. The combination of these two effects was a small increase in *K*_*D*_ (Table 3). It is also worth emphasising, however, that very small changes in kinetic parameters may also be a consequence of the need to fit association curves for four or more concentrations of fluorescent ligand simultaneously with shared values for *k*_on_ and *k*_off_.

To explore further the influence of receptor phosphorylation, we generated a tyrosine phosphorylation‐deficient VEGFR2 (VEGFR2‐TPD) where key intracellular phosphotyrosine residues were mutated to phenylalanine (Y951F, Y1054F, Y1059F, Y1175F, and Y1214F; Figure 4d). This mutant form of VEGFR2 was unable to stimulate NFAT signalling but mimicked the effect of cediranib on VEGF‐TMR binding and produced a significant increase in the amplitude of the NanoBRET binding signal obtained with 20‐nM VEGF_121_a‐TMR. This is likely to be due to interference with endocytosis pathways that are dependent on agonist‐induced VEGFR2 activation and phosphorylation. However, what is clear from both the experiments with VEGFR2‐TPD and cediranib is that additional pathways can mediate VEGFR2 endocytosis. For example, endocytosis of both VEGFR2‐TPD and VEGF_121_a‐TMR was still observed using confocal microscopy (Figure 4).

Our data suggest that substantial agonist‐induced VEGFR2 endocytosis is occurring in intact HEK293T cells over the concentration range (1–20 nM) and timecourse (20 min) used previously to assess ligand‐binding kinetics in live cells (Kilpatrick et al., 2017; Peach, Kilpatrick, et al., 2018). Furthermore, this internalisation could not be prevented by interfering with agonist‐induced VEGFR2 phosphorylation. To investigate the impact that agonist‐induced endocytosis (or association with other signalling complexes and ancillary proteins in living cells) may have on the estimation of kinetic ligand‐binding parameters deduced by NanoBRET in intact cells, we established an isolated membrane ligand‐binding assay where the potential for parallel agonist‐induced receptor endocytosis and engagement with cytoplasmic signalling complexes would be disrupted. NanoBRET ligand binding with all three fluorescent VEGF‐A isoforms demonstrated clear specific binding in isolated membrane preparations. Non‐specific binding, determined in the presence of a high concentration of non‐fluorescent VEGF‐A ligand, was low over the full concentration range of fluorescent ligand employed. The equilibrium *K*_*D*_ values determined following a 1‐hr incubation were slightly more potent than those previously reported in intact cells (Table 2; Kilpatrick et al., 2017; Peach, Kilpatrick, et al., 2018). This was particularly the case for VEGF_165_a‐TMR. Similarly, VEGF‐Ax produced competitive inhibition of the binding of each fluorescent VEGF‐A isoform in membrane preparations. However, in marked contrast to equivalent studies in intact living cells, the kinetics of the binding of five different concentrations of VEGF_165_a‐TMR, VEGF_165_b‐TMR, or VEGF_121_a‐TMR produced classic ligand‐binding association curves that were maintained for the duration of the 90‐min experiment.

The *k*_on_ and *k*_off_ values determined in membrane preparations were of a similar order to those previously reported for these three fluorescent ligands in intact cells when the kinetic analysis was restricted to the first 20 min of agonist stimulation, in order to reduce the influence of receptor endocytosis (Peach, Kilpatrick, et al., 2018). *k*_on_ rate constants were slightly smaller (1.2‐fold to 2.0‐fold) in membranes than the corresponding values obtained in intact cells. However, *k*_off_ rate constants were approximately fourfold lower (Table 2) than those determined in HEK293T cells, indicative of a slower agonist dissociation rate. In matched experiments (Figure 6 and Table 3), these differences were significant (*P* < .05). These data suggest that the impact of agonist‐induced VEGFR2 endocytosis on kinetically derived *k*_on_, *k*_off_, and *K*_*D*_ values is not large if the analysis in cells is restricted to early time points. However, the process of receptor endocytosis does lead to an underestimation of the equilibrium off‐rate kinetic constant and receptor residence time (1/*k*_off_) of VEGF_165_b‐TMR on the receptor at the plasma membrane (e.g. the calculated residence time in cells for VEGF_165_b‐TMR was 12.3 min whilst that in membranes preparations was 43.5 min; Table 3). This is consistent with our earlier suggestion that rapid dissociation of VEGF_165_a‐TMR from VEGFR2 occurs in the environment of intracellular endosomes (as a consequence of the lower pH or the presence of endosomal proteases) and allows rapid recycling of ligand‐free VEGFR2 back to the cell surface (Kilpatrick et al., 2017). Interestingly, in the tyrosine phosphorylation‐deficient VEGFR2‐TPD, the kinetic constants determined in membranes for VEGF_121_a‐TMR were within a factor of two of those derived in membranes for the wild‐type receptor.

The ability to define the pH at which ligand‐binding studies are undertaken in membrane preparations provided an opportunity to study the influence of pH on ligand binding at the acidic pH normally found in intracellular endosomes. These data showed that the association and dissociation rate constants of fluorescent VEGF‐A at NanoLuc‐VEGFR2 were faster at pH 6.0 than those observed at pH 7.4. Indeed, the parameters approached those obtained in intact cells (Table 3). These data suggest that a proportion of the ligand‐binding characteristics observed in intact cells is a consequence of receptor endocytosis and the influence of the lower pH environment.

In summary, the present study has shown for the first time that NanoBRET can be used to monitor the kinetics of the binding of fluorescent VEGF‐A isoforms to VEGFR2 in isolated membrane preparations. Equilibrium measurements in membranes produced binding parameters that were of a similar order to those determined in live cells. However, in contrast to previous studies in intact cells where the NanoBRET signal falls towards baseline values after reaching a peak, kinetic experiments in membranes produced classic ligand‐binding association curves that were maintained for the duration of the 90‐min experiment. Automated imaging allowed a quantitative analysis of the effect of fluorescent VEGF‐A isoforms on VEGFR2 endocytosis in intact cells. These studies confirmed that all three fluorescent ligands produced a rapid and potent translocation of ligand‐bound VEGFR2 to intracellular endosomes.

Our data suggest that the largest impact of this rapid agonist‐induced VEGFR2 endocytosis on ligand‐binding parameters was on the equilibrium off‐rate kinetic constant (*k*_off_) and receptor residence time (1/*k*_off_). Thus, rapid VEGFR2 endocytosis into intracellular endosomes receptor in intact cells shortened the measured residence time of VEGF_165_b‐TMR on the receptor from 43.5 min (in membranes) to 12.3 min (in cells). These data suggest that the ligand‐binding kinetics of VEGF‐A isoforms differ between plasma membrane and intracellular endosomes and that agonist‐induced receptor endocytosis can change both local signalling environment and ligand‐binding kinetic properties of the receptor. These data provide important new insights into the impact of cellular location and pH on the kinetics of ligand–receptor interactions for a receptor that is a key mediator of both angiogenesis and vascular permeability, and an important drug target for the treatment of cancer.

## CONFLICT OF INTEREST

The authors declare no conflicts of interest.

## AUTHOR CONTRIBUTIONS

S.J.H., J.W., L.E.K., and C.J.P. conceived the study; C.J.P., L.E.K., S.J.H., and J.W. participated in the research design; C.J.P. conducted the experiments; C.J.P. and S.J.H. performed the data analysis; and C.J.P., L.E.K., J.W., and S.J.H. wrote or contributed to the writing of the manuscript.

## DECLARATION OF TRANSPARENCY AND SCIENTIFIC RIGOUR

This Declaration acknowledges that this paper adheres to the principles for transparent reporting and scientific rigour of preclinical research as stated in the *BJP* guidelines for Design & Analysis and Immunoblotting and Immunochemistry and as recommended by funding agencies, publishers, and other organisations engaged with supporting research.
